# Alterations in Source-Sink Relations Affect Rice Yield Response to Elevated CO_2_: A Free-Air CO_2_ Enrichment Study

**DOI:** 10.3389/fpls.2021.700159

**Published:** 2021-07-02

**Authors:** Bo Gao, Shaowu Hu, Liquan Jing, Xichao Niu, Yunxia Wang, Jianguo Zhu, Yulong Wang, Lianxin Yang

**Affiliations:** ^1^Key Laboratory of Crop Genetics and Physiology of Jiangsu Province, Yangzhou University, Yangzhou, China; ^2^Jiangsu Co-Innovation Center for Modern Production Technology of Grain Crops of Jiangsu Province, Yangzhou University, Yangzhou, China; ^3^Jiangsu Coastal Area Institute of Agricultural Sciences, Yancheng, China; ^4^College of Environmental Science and Engineering, Yangzhou University, Yangzhou, China; ^5^State Key Laboratory of Soil and Sustainable Agriculture, Institute of Soil Science, Chinese Academy of Sciences, Nanjing, China

**Keywords:** climate change, free-air CO_2_ enrichment, *Oryza sativa*, source-sink ratio, yield, photosynthesis

## Abstract

To understand the effects of source-sink relationships on rice yield response to elevated CO_2_ levels (eCO_2_), we conducted a field study using a popular *japonica* cultivar grown in a free-air CO_2_ enrichment environment in 2017–2018. The source-sink ratio of rice was set artificially via source-sink treatments (SSTs) at the heading stage. Five SSTs were performed in 2017 (EXP1): cutting off the flag leaf (LC1) and the top three functional leaves (LC3), removing one branch in every three branches of a panicle (SR1/3) and one branch in every two branches of a panicle (SR1/2), and the control (CK) without any leaf cutting or spikelet removal. The eCO_2_ significantly increased grain yield by 15.7% on average over all treatments; it significantly increased grain yield of CK, LC1, LC3, SR1/3, and SR1/2 crops by 13.9, 18.1, 25.3, 12.0, and 10.9%, respectively. The yield response to eCO_2_ was associated with a significant increase of panicle number and fully-filled grain percentage (FGP), and the response of crops under different SSTs was significantly positively correlated with FGP and the average grain weight of the seeds. Two SSTs (CK and LC3) were performed in 2018 (EXP2), which confirmed that the yield response of LC3 crops (25.1%) to eCO_2_ was significantly higher than that of CK (15.9%). Among the different grain positions, yield response to eCO_2_ of grains attached to the lower secondary rachis was greater than that of grains attached to the upper primary rachis. Reducing the source-sink ratio via leaf-cutting enhanced the net photosynthetic rate response of the remaining leaves to eCO_2_ and increased the grain filling ability. Conversely, spikelet removal increased the non-structural carbohydrate (NSC) content of the stem, causing feedback inhibition and photosynthetic down-regulation. This study suggests that reducing the source-sink ratio by adopting appropriate management measures can increase the response of rice to eCO_2_.

## Introduction

Anthropogenic activities including fossil fuel burning and deforestation, have increased the level of atmospheric carbon dioxide (CO_2_) from about 280 ppm (Intergovernmental Panel on Climate Change [IPCC], 2007) during the Industrial Revolution to a current 410 ppm (National Oceanic and Atmospheric Administration [NOAA], 2020), which is projected to exceed 580 ppm (Intergovernmental Panel on Climate Change [IPCC], 2013) by 2050. As the substrate of photosynthesis, the increasing CO_2_ level has a “fertilizer effect” on plant growth (Ainsworth and Long, 2005). Rice is the staple food for more than half of the global population (Fan et al., 2016). Fertilization and genetic modification have led an increase in rice yields; however, due to negative factors such as increasing ozone concentrations, global warming, and drought stress, the growth rate of global rice yield has been slowing down since the beginning of this century (Ainsworth, 2008; Long, 2012). According to the Food and Agriculture Organization, an additional 30% of rice production is required to meet the demands of a global population of more than 9 billion by 2050 (Food and Agriculture Organization [FAO], 2009; Alexandratos and Bruinsma, 2012). Therefore, it is important to use the ‘fertilizer effect’ of elevated CO_2_ (eCO_2_) to maximize the potential of rice yield for ensuring food security.

Long et al. (2006), using a theoretical model, pointed out that an increasing CO_2_ concentration to 550 ppm at 25°C could increase the leaf net photosynthetic rate (*Pn*) of C_3_ crops by about 38%. However, free-air CO_2_ enrichment (FACE) experiments have indicated that the *Pn* was only increased by 20%, with even lower increases in biomass (17%) and final yield (13%). This indicates that the source-sink relationship of the crop population under eCO_2_ is far from the theoretical model, with a limited response of rice yield under eCO_2_. In this case, C_3_ crops may not be able to efficiently use eCO_2_ in the future (Sonnewald and Fernie, 2018). A recent meta-analysis (Hu et al., 2021) of FACE studies suggests that there are significant genotypic differences in yield increase under eCO_2_. The yield enhancement by eCO_2_ of *hybrid* rice (24.7%) is significantly greater than that of conventional rice (14.2%). This is mainly because of the large sink capacity (more spikelets per panicle and unit area) and adaptive plasticity of *hybrid* rice (Dingkuhn et al., 2020), indicating that the sink capacity of rice plays an important role in the response to increasing CO_2_ levels. By integrating the FACE studies involving seven rice varieties, Ainsworth and Long (2021) found a significant positive correlation between yield increase and yield potential [the product of spikelet number per unit area (SNA) and single grain weight] when the CO_2_ concentration was increased by 200 ppm. Similarly, by conducting a pot experiment with five naturally contrasting cultivars in the source-sink ratio and grown in a greenhouse under controlled conditions, Fabre et al. (2020) found that the response of *Pn* of cultivars with a low source-sink ratio to eCO_2_ was greater than that with a high source-sink ratio. Yield, biomass, and seed setting traits showed a similar trend, but these parameters were not statistically significant. These results indicate that under eCO_2_, the yield increase potential of rice does not reach the theoretical expectation and is closely related to the source-sink relationship. However, there are few reports on the response of rice yield to eCO_2_ by adjusting the ratio of source or sink, especially under field conditions.

Cutting off leaves or removing spikelets are commonly used methods to study the source-sink relationship of crops. The original source-sink relationship of rice could be artificially changed by reducing the source and/or sink at the heading stage and then compared with the control plants in these methods. A few studies have used this method to investigate the interaction between the source-sink relationship and eCO_2_. For instance, by conducting a chamber study using the *japonica* cultivar Kirara397, Shimono et al. (2010) found that eCO_2_ significantly reduced *Pn* of CK and spikelet-removal (SR) crops by 23 and 37% at the mid-grain filling stage, respectively, when compared with ambient conditions. The SR reduced *Pn* under eCO_2_, compared with CK, but it showed an opposite trend under ambient conditions. Conversely, Fabre et al. (2019) observed that SR significantly reduced the *Pn* of the *indica* cultivar IR64 at 2 weeks after heading compared with the CK under ambient conditions. This phenomenon was more obvious under eCO_2_, suggesting that SR had a certain inhibitory effect on *Pn* compared with CK. Recently, a FACE experiment (Zhang et al., 2019) showed that reducing the source-sink ratio of the *japonica* cultivars Wuyunjing 23 and Nanjing 9108 by cutting off the second and third leaves did not negate the photosynthetic downregulation of the flag leaf under eCO_2_. However, effects of the interaction between leaf-cutting (LC) and eCO_2_ on yield traits were not observed in these studies. To date, there is no systematic report about the impact of LC or SR on rice yield response to eCO_2_ under open-paddy conditions.

The filling capacity and weight traits of the grain are closely related to the location of the rice panicle (Wang et al., 1995; Yang et al., 2000). Generally, the spikelets located on the primary apical branches of the rice panicle develop and blossom early and are known as superior spikelets (SS). The spikelets located on the proximal secondary branches develop and blossom late and are known as inferior spikelets (IS). Previous studies have shown that the sensitivity to environmental changes differs between SS and IS, with IS being affected more significantly by changes in environmental conditions, such as drought (Dong et al., 2014) and ozone stress (Shao et al., 2020). Zhang et al. (2013, 2015) found that, in a FACE trial, eCO_2_ increased IS grain weight but had no significant effect on SS. However, the impacts of the interactions between CO_2_ and LC or SR on yield and its components of different grain positions remain unknown.

Because of the disturbance to the microclimate around the plants and the limited space (McLeod and Long, 1999), the chamber is not suitable to study the interactions between source-sink and CO_2_ at the population level. The FACE system, developed at the end of the 20th century, adopts standard crop management techniques to study rice performance at the field population level with free airflow, providing an opportunity to explore the rice source-sink relationship (Long et al., 2006; Yoshinaga et al., 2020). To investigate the effects and possible reasons of artificial source-sink treatments (SSTs) on the response of rice yield to eCO_2_, we grew the super *japonica* cultivar Wuyunjing 27, which is widely used in production, under an eCO_2_ environment in a FACE facility in 2017–2018. We constructed rice plants with gradient differences in source-sink levels by cutting off leaves or removing spikelets at the heading stage for comparison with naturally growing rice. The objectives of this study were to (1) identify the effects of eCO_2_ on yield and its components, biomass accumulation and distribution, non-structural carbohydrate (NSC) contents of the stem, and *Pn* of leaves located in different positions under different source-sink levels, (2) investigate the differences between SS and IS on yield traits under eCO_2_ and SSTs, and (3) investigate the mechanisms of adjustments by LC and SR in rice yield response to eCO_2_ from the perspectives of growth and photosynthesis.

## Materials and Methods

### Experimental Site and FACE System

The FACE system was established in Yangzhou (119°42′0′′E, 32°35′5′′N), Jiangsu Province, China. Detailed descriptions of the FACE platform are available elsewhere (Yang et al., 2009; Jing et al., 2021). Briefly, the FACE system had six plots located in different paddies with similar soil and agronomic histories. Three plots were randomly allocated for the elevated CO_2_ treatments and three for the ambient CO_2_. Each FACE plot was about 80 m^2^, and the distance between the center of the FACE and ambient plots was 90 m to avoid contamination of ambient conditions by CO_2_. Pure CO_2_ gas was emitted into the center through pipelines installed around the FACE plots. The CO_2_ concentration of the platform was monitored and controlled via a computer network. The release speed and direction of CO_2_ gas were automatically adjusted to match the atmospheric CO_2_ concentration, wind direction, wind speed, and CO_2_ concentration at the crop canopy height to maintain the CO_2_ concentration in the main growth period of rice in the FACE plots at 200 ppm higher than the ambient concentration. The CO_2_ treatment began after seedling transplanting and continued until plant maturity. Treatment was performed daily from sunrise to sunset. The average temperatures from July 01 to October 31 were 24.9 and 25.3°C in 2017 and 2018, respectively (Supplementary Figures 1A,B).

### Crop Cultivation

The *japonica* super rice cultivar Wuyunjing 27 (WYJ27), a popular cultivar in the study region, was selected. Seeds were sown on May 22 of 2017 and 2018 and grown under ambient air for 30 days. Seedlings were manually transplanted to all plots at a density of two seedlings per hill on June 21 in both growing seasons. Hill spacing was 16.7 cm × 25 cm (equivalent to 24 hills m^–2^). Harvesting was performed on October 13 in 2017 and on October 17 in 2018. Nitrogen was applied as urea (*N* = 46%) and compound chemical fertilizer (N: P_2_O_5_: K_2_O = 15:15:15) at a rate of 22.5 g N m^–2^. In each growing season, nitrogen was applied in three occasions (40% of the total as a basal dressing 1 day before transplanting, 30% as a top dressing at the early tillering stage, and 30% as a top dressing at panicle ignition). The P and K were applied as compound fertilizer P_2_O_5_ and K_2_O at a rate of 9 g m^–2^ 1 day before transplanting. For water management, the paddy fields were submerged in water at 5 cm from June 17 to July 20 and then subjected to wet-dry cycles through natural drainage and intermittent irrigation from July 21 to August 10 for both years. Diseases and insects were monitored and controlled during the growing season. Fertilizer application and water management are described elsewhere (Zhao et al., 2019).

### Source-Sink Treatment

Before the SST, plants with the same growth potential (according to the average number of tillers) in each plot were selected and labeled at the heading stage. In 2017 (EXP1), five SSTs were set by cutting off the flag leaf (LC1) and the top three functional leaves (LC3), removing one branch in every three branches of a panicle (SR1/3) and one branch in every two branches of a panicle (SR1/2); in the control (CK), there was no leaf-cutting (LC) or spikelet-removal (SR). In 2018 (EXP2), two SSTs were set by LC3 and CK. A total of 15 hills were selected for the source–sink manipulation within each plot in both EXP1 and EXP2.

### Grain Yield and Biomass

At DAT10 (DAT, day after SST), DAT20, and DAT35, three representative hills (based on the average tiller number per hill) were destructively sampled. Samples were separated into leaves, stems (including leaf sheaths), and panicles, and oven-dried at 105°C for 30 min and at 80°C for 72 h. At maturity, five hills in each treatment were selected to determine grain yield and dry matter weight. The panicles per hill were counted to compute the panicles per unit area. Stems and leaves were oven-dried at 105°C for 30 min, followed by 80°C for 72 h. Panicles were air-dried to maintain a constant weight. The stems, leaves, and panicles were weighed to determine dry matter weight. The panicles were hand-threshed, and filled grains, incomplete-filled grains, and empty grains were separated using a winnowing machine (FX-II, Huier Ltd., Hangzhou, China). In 2018, the panicle grains were categorized into three groups according to their position on the panicle, using the following procedure (Finkelstein, 2010; Shao et al., 2020): The panicle was equally divided into upper and lower parts with the same number of primary branches (when the number of primary branches was odd, the upper part had one more branch than the lower part). Grains on the primary branches of the upper part of the panicle were referred to as the superior spikelet (SS), whereas grains on the secondary branches of the lower part represented the inferior spikelet (IS). The remaining grains of the panicle were referred to as the medium spikelet (MS). Grain number and grain weight were determined. Yield and its traits were calculated as follows: FGP (%) = fully-filled grain number × 100/total spikelet number; incomplete-filled grain percentage (IGP) (%) = incomplete-filled grain number × 100/total spikelet number; empty grain percentage (EGP) (%) = empty grain number × 100/total spikelet number; fully-filled grain weight (FGW) = total FGW (mg)/fully-filled grain number, average grain weight of all seeds (AGW) = weight of all seeds/number of all seeds, and yield (g m^–2^) = panicle number per m^2^ × spikelet number per panicle (SNP) × FGP × FGW (mg)/1,000.

### Leaf Net Photosynthetic Rate (*Pn*) Measurement

A portable photosynthesis system (Li-6400, Li-COR Inc., United States) was used to determine the leaf *Pn* between 9:00–11:30 and 14:30–16:30 on sunny days. Two plants of each treatment in each plot were selected for determination at DAT10, DAT20, and DAT35. The flag leaf, the second leaf, and the fourth leaf were measured for CK rice. The flag leaf was measured for SR1/3 and SR1/2 rice. The second leaf and the fourth leaf were measured for LC1 and LC3 rice, respectively. The measuring position was between 1/2 and 1/3 of the tip of the leaf. The instrument was properly connected and calibrated, and the Li-COR injection system was used to control the CO_2_ concentration. The CO_2_ concentrations in ambient and FACE plots were set at 380 and 580 ppm, respectively. The red and blue light source was selected, and the light intensity was set according to the natural light source at each measurement stage (1,200, 1,000, and 1,000 μmol m^–2^ s^–1^ at DAT10, DAT20, and DAT35, respectively). Air temperature of the measurement cuvette was maintained at 30°C, with a relative humidity of 65%. The measured leaves were kept for about 100 s in the leaf chamber to reach a stable photosynthesis state before counting.

### Non-structural Carbohydrate (NSC) Content Analysis

The NSC content was determined by the sum of total soluble sugar and starch. The anthrone H_2_SO_4_ method (Yoshida et al., 1976) was used to determine the soluble sugar and starch contents of the stem and leaf. In detail, the oven-dried stem and leaf samples were ground by a pulverizer, sieved through a 100-mesh sieve, and 50 mg per sample was added into 10-mL centrifuge tubes. Subsequently, 5 mL 80% ethanol was added to the tube, which was placed in a water bath at 80°C for 30 min. The samples were then centrifuged at 4,000 rpm for 10 min before supernatant extraction. These steps were repeated three times; 0.1 g activated carbon was added to the supernatant, which was then left to stand for 12 h at room temperature. The solution was then filtered and transferred to a 50-mL volumetric flask, diluted to volume, and mixed. The residue was dried for the measurement of starch. To determine total soluble sugars, 1.0 mL of the extract was absorbed, and 5 mL 0.2% anthrone reagent was added; the mixture was shaken well and boiled in a water bath for 15 min. Subsequently, it was cooled to room temperature, colorimetry was performed at a wavelength of 620 nm, and the OD value was recorded. The soluble sugar content in the extract was calculated using the standard curve of pure glucose analysis. For the determination of starch, 1.0 mL distilled water was added to the residue, which was then boiled in a water bath for 20 min, with continuous stirring. Subsequently, 1.0 mL of 9.2 mol L^–1^ HCLO_4_ was added after cooling while shaking for 10 min. Water (3 mL) was added, and the mixture was centrifuged at 4,000 rpm for 15 min. Then, 4.6 mol L^–1^ HCLO_4_ was added to the residue, and the supernatant was extracted. The supernatant was combined and diluted to a constant volume of 50 mL. The extracted glucose was determined using the method for soluble sugar and then converted into starch.

### Statistical Analysis

The field experiment was a completely randomized design with a split-plot arrangement. The CO_2_ was treated as the main plot, and SSTs were subplots with three replications. Analysis of variance (ANOVA) was performed using the SPSS statistical software (SPSS 20.0, SPSS Inc., Chicago, IL, United States). The least significant difference was used to compare the means among treatments. Statistically significant effects were indicated as follows: ^∗∗^*P* < 0.01, ^∗^*P* < 0.05, ^+^*P* < 0.1, and not statistically significant (ns) *P* ≥ 0.1. Pearson’s correlations were calculated to determine the relationships among the different parameters of yield and its components.

## Results

### Grain Yield and Components of the Whole Panicle

**FIGURE 1:**
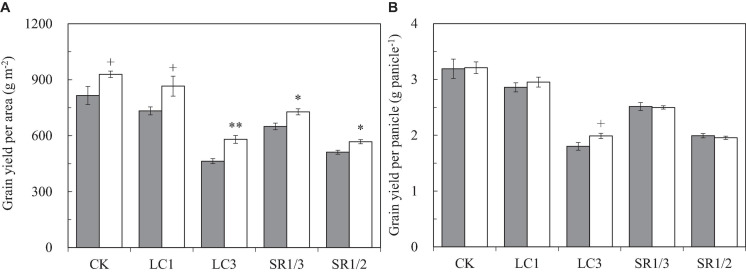
Grain yield per area **(A)** and grain yield per panicle **(B)** of WYJ27 affected by elevated CO_2_ and different source-sink treatments in 2017. Each bar in the figure represents the mean values across three plots for ambient CO_2_ (aCO_2_, filled square) or elevated CO_2_ (eCO_2_, aCO_2_ + 200 ppm, unfilled square); vertical bars represent standard error (*n* = 3). CK, no leaf cutting or spikelet removing; LC1, cutting off the flag leaf; LC3, cutting off top three leaves; SR1/3, removing one branch in every three branches of a panicle; SR1/2, removing one branch in every two branches of a panicle. **P < 0.01, *P < 0.05, ^+^*P* < 0.1.

Compared with ambient CO_2_, eCO_2_ significantly increased the grain yield per unit area (GYA) of WYJ27 by 15.7% on average, and crop yields under CK, LC1, LC3, SR1/3, and SR1/2 treatments were significantly increased by 13.9, 18.1, 25.3, 12.0, and 10.9%, respectively (Figure 1A). Compared with CK, the LC1, LC3, SR1/3, and SR1/2 treatments significantly reduced GYA by 8.3, 40.1, 21.0, and 38.1%, respectively. Grain yield per panicle (GYP) was obtained by dividing GYA by panicle number per unit area (PNA), which showed a similar trend to GYA in the response to eCO_2_, LC, and SR. Compared with CK, LC3 significantly increased the response of GYP to eCO_2_, whereas SR caused a slight reduction (Figure 1B). No significant interaction effect between CO_2_ and SSTs was observed on GYA or GYP.

**FIGURE 2:**
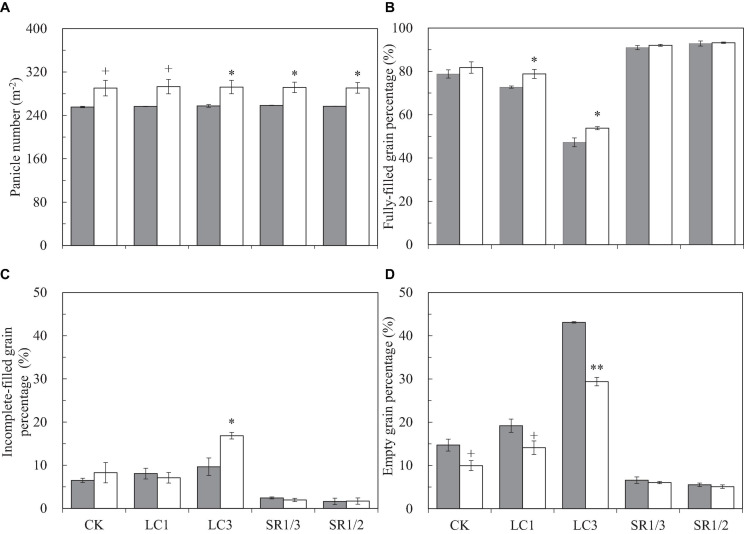
Panicle number per area **(A)**, fully-filled grain percentage **(B)**, incomplete-filled grain percentage **(C)**, empty grain percentage **(D)** of WYJ27 affected by elevated CO_2_ and different source-sink treatments in 2017. Each bar in the figure represents the mean values across three plots for ambient CO_2_ (aCO_2_, filled square) or elevated CO_2_ (eCO_2_, aCO_2_ + 200 ppm, unfilled square); vertical bars represent standard error (*n* = 3). CK, no leaf cutting or spikelet removing; LC1, cutting off the flag leaf; LC3, cutting off top three leaves; SR1/3, removing one branch in every three branches of a panicle; SR1/2, removing one branch in every two branches of a panicle. **P < 0.01, *P < 0.05, ^+^*P* < 0.1.

We observed that eCO_2_ did not affect SNP but significantly increased PNA and total SNA by 13.5 and 12.3%, respectively (Figure 2A and Supplementary Figures 2A,B). Compared with CK, LC had no significant effect on the above three parameters, but SNP and SNA were significantly reduced by SR treatments: SR1/3 and SR1/2 significantly reduced SNP by 34.9 and 51.4%, respectively, and the reduction of SNA was almost the same with SNP. No significant interaction effect between CO_2_ and SSTs was observed on these three parameters.

To better understand the grain filling capacity, we measured not only FGP but also IGP and EGP. Compared with ambient CO_2_, eCO_2_ significantly increased FGP by 4.4% on average, and crop yields under CK, LC1, LC3, SR1/3, and SR1/2 were increased by 3.8, 8.3^∗^, 13.8^∗^, 1.1, and 0.4%, respectively (Figure 2B). On average, eCO_2_ slightly increased IGP (Figure 2C) but significantly decreased EGP by 27.6% (Figure 2D) compared with ambient CO_2_. Among the crops under different SSTs, eCO_2_ significantly decreased the EGP of CK, LC1, and LC3 crops by 32.4, 26.6, and 31.8%, respectively, whilst no changes were observed in SR crops. Compared with CK, LC1 and LC3 significantly reduced FGP by 5.6 and 37.1%, respectively, and SR1/3 and SR1/2 significantly increased FGP by 13.9 and 15.9%, respectively. The reduction of FGP caused by LC was mainly related to the increase of EGP, whereas the significant increase in FGP in SR crops was mainly related to the decrease of both EGP and IGP. Variance analysis showed that CO_2_ × LC3, CO_2_ × SR1/3, and CO_2_ × SR1/2 had different degrees of interaction on EGP (Table 1).

**TABLE 1:** Significance test for yield, yield traits, biomass per area, and dry matter distribution of WYJ27 under elevated CO_2_ and different source-sink treatments in 2017.

Parameters	CO_2_	LC1	LC3	SR1/3	SR1/2	CO_2_ × LC1	CO_2_ × LC3	CO_2_ × SR1/3	CO_2_ × SR1/2
Grain yield per area	**↑	+↓	**↓	**↓	**↓	ns	ns	ns	ns
Grain yield per panicle	ns	*↓	**↓	**↓	**↓	ns	ns	ns	ns
Panicle number per unit area	**↑	ns	ns	ns	ns	ns	ns	ns	ns
Spikelet number per panicle	ns	ns	ns	**↓	**↓	ns	ns	ns	ns
Spikelet number per unit area	**↑	ns	ns	**↓	**↓	ns	ns	ns	ns
Fully-filled grain percentage	**↑	*↓	**↓	**↑	**↑	ns	ns	ns	ns
Incomplete-filled grain percentage	+↑	ns	**↑	**↓	**↓	ns	ns	ns	ns
Empty grain percentage	**↓	*↑	**↑	**↓	**↓	ns	**	+	+
Fully-filled grain weight	*↓	ns	ns	**↑	**↑	ns	ns	ns	ns
Average grain weight of all seeds	ns	ns	**↓	**↑	**↑	ns	ns	ns	ns
Aboveground biomass per unit area	**↑	+↓	**↓	ns	+↓	ns	ns	ns	ns
Stem biomass per unit area	**↑	ns	**↓	**↑	**↑	ns	ns	ns	ns
Leaf biomass per unit area	**↑	**↓	**↓	ns	*↑	ns	ns	ns	ns
Panicle biomass per unit area	**↑	+↓	**↓	**↓	**↓	ns	ns	ns	ns
Ratio of stem	*↑	**↑	**↑	**↑	**↑	ns	*	ns	*
Ratio of leaf	*↓	**↓	**↓	*↑	**↑	ns	ns	ns	ns
Ratio of panicle	ns	ns	**↓	**↓	**↓	ns	*	ns	*

LC1, cutting off the flag leaf; LC3, cutting off top three leaves; SR1/3, removing one branch in every three branches of a panicle; SR1/2, removing one branch in every two branches of a panicle. **P < 0.01, *P < 0.05, ^+^*P* < 0.1; ns, not significant; *n* = 3. Arrows in the treatment column indicate treatment increased (↑) or decreased (↓) the values.

Unlike the three afore-mentioned parameters, the responses of grain weight to eCO_2_ and SSTs were small (Supplementary Figures 2C,D). Both eCO_2_ and LC had little effect on the FGW (ca.1%). However, compared with CK, SR1/3 and SR1/2 significantly increased FGW by 5.6 and 9.4%, respectively. The AGW was the mean grain weight of all grains, including fully-filled grains, unfilled grains, and empty grains; the response of AGW to eCO_2_ and SSTs was similar to that of FGP, but the response range was significantly smaller.

Correlation analysis showed that the response of GYA to eCO_2_ had no significant correlation with the responses of PNA, SNP, SNA, FGW, EGP, and IGP (Table 2), but was significantly positively correlated with FGP (*r* = 0.994^∗∗^, Supplementary Figure 3A) and AGW (*r* = 0.976^∗∗^, Supplementary Figure 3B). The response of GYP to eCO_2_ was not significantly correlated with SNP and FGW, but it was positively correlated with FGP (*r* = 0.981^∗∗^, Supplementary Figure 3C) and AGW (*r* = 0.962^∗∗^, Supplementary Figure 3D).

**TABLE 2:** Relationships among the CO_2_-induced changes in yield, yield traits of five source-sink treats under elevated CO_2_.

Index		GY		PNA	SNP	SNA	FGP	IGP	EGP	FGW	AGW
		GYA	GYP								
GY	GYA	1									
	GYP	0.996**	1								
PNA		0.493	0.417	1							
SNP		0.438	0.508	–0.279	1						
SNA		0.739	0.770	0.248	0.859^+^	1					
FGP		0.994**	0.981**	0.581	0.372	0.718	1				
IGP		0.746	0.782	0.150	0.843^+^	0.944*	0.709	1			
EGP		–0.713	–0.692	–0.726	–0.429	−0.811^+^	–0.755	–0.648	1		
FGW		−0.872^+^	−0.847^+^	–0.755	–0.388	–0.802	−0.905*	–0.719	0.951*	1	
AGW		0.976**	0.962**	0.511	0.265	0.581	0.976**	0.589	–0.617	−0.801	1

(Pearson correlation matrix). GY, grain yield; GYA, grain yield per area; GYP, grain yield per panicle; PNA, panicle number per area; SNP, spikelet number per panicle; SNA, spikelet number per area; FGP, fully-filled grain percentage; IGP, incomplete-filled grain percentage; EGP, empty grain percentage; FGW, fully-filled grain weight; AGW, average grain weight of all seeds; **P < 0.01, *P < 0.05, ^+^*P* < 0.1. *n* = 5.

### Grain Yield and Components of Different Grain Positions

**FIGURE 3:**
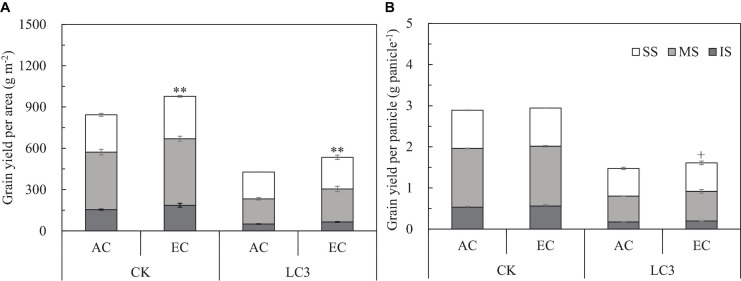
Grain yield per area **(A)** and grain yield per panicle **(B)** of WYJ27 affected by elevated CO_2_ and leaf-cutting treatment in 2018. Each bar in the figure represents the mean values across three plots for ambient CO_2_ (AC) or elevated CO_2_ (EC, AC + 200 ppm); vertical bars represent standard error (*n* = 3). CK, no leaf cutting; LC3, cutting off top three leaves. P, grain position; SS, superior spikelet; MS, medium spikelet; IS, inferior spikelet. **P < 0.01, ^+^*P* < 0.1.

We measured yield and its components for grains located at different panicle positions of CK and LC3 crops in 2018 (EXP2). Compared with ambient CO_2_, eCO_2_ significantly increased the GYA by about 19.0% (Figure 3A); among the different SSTs, the CK and LC3 crops were significantly increased by 15.9 and 25.1%, respectively. In terms of grain position, eCO_2_ increased the yield of SS, MS, and IS by 15.6, 20.4^∗∗^, and 22.7%^∗∗^, respectively. Compared with CK, LC3 significantly decreased GYA by 47.2% on average. Among the different grain positions, LC3 significantly decreased the GYA of SS, MS, and IS by 26.7, 53.2, and 66.1%, respectively. The response trend of GYP to eCO_2_ was similar to that of GYA (Figure 3B): the response of LC3 crops (9.1%) was significantly higher than that of CK (1.9%); the responses of MS (5.6%) and IS (7.3%) were higher than that of SS (1.3%) on average for two factors (CO_2_ and SSTs). Position × LC3 had a significant interaction effect on GYP and GYA (Table 3).

**TABLE 3:** Significance test for grain yield per area and per panicle of WYJ27 under elevated CO_2_ and different source-sink treatments in 2018.

Parameters	CO_2_	LC3	P	CO_2_ × LC3	CO_2_ × P	LC3 × P	CO_2_ × LC3 × P
Grain yield per area	**↑	**↓	**	ns	ns	**	ns
Grain yield per panicle	*↑	*↓	**	ns	ns	**	ns

LC3, cutting off top three leaves. P, grain position; SS, superior spikelet; MS, medium spikelet; IS, inferior spikelet. **P < 0.01, *P < 0.05; *n* = 3. Arrows in the treatment column indicate treatment increased (↑) or decreased (↓) the values.

Compared with ambient CO_2_, eCO_2_ did not affect SNP but significantly increased PNA and SNA by 14.0% on average (Table 4). The LC3 had no significant effect on the above three parameters compared with CK. We observed no significant interaction effect between CO_2_, LC3, and position on the three parameters.

**TABLE 4:** Panicle number per area, spikelet number per panicle and total spikelet number per area, filled grain percentage, incomplete-filled grain percentage, empty grain percentage, fully-filled grain weight, average grain weight of all seeds of WYJ27 as affected by elevated CO_2_, grain position and leaf-cutting treatment in 2018.

LC	CO_2_	Panicle number (m^–2^)	Spikelet number (panicle^–1^)			Total spikelet number (10^3^ m^–2^)			Filled grain percentage (%)			Incomplete-filled grain percentage (%)			Empty grain percentage (%)			Fully-filled grain weight (mg)			Average grain weight of all seeds (mg)		
			SS	MS	IS	SS	MS	IS	SS	MS	IS	SS	MS	IS	SS	MS	IS	SS	MS	IS	SS	MS	IS
CK	AC	292.1	36.0	68.6	39.3	10.5	20.0	11.5	96.6	84.1	62.9	0.6	9.8	17.1	2.9	6.1	20.1	26.7	24.8	21.5	25.9	22.2	16.2
	EC	332.0	35.8	67.0	39.4	11.9	22.2	13.1	96.2	86.9	64.6	1.0	6.1	19.3	2.8	7.0	16.1	27.0	25.0	21.9	26.2	22.7	16.6
LC3	AC	290.4	37.3	67.7	40.2	10.8	19.7	11.7	75.2	39.4	20.7	6.0	11.5	13.6	18.8	49.1	65.8	24.0	23.5	20.9	19.5	12.2	7.2
	EC	332.0	35.7	69.0	42.2	11.8	22.9	14.0	79.3	43.6	21.2	10.5	12.9	17.1	10.2	43.5	61.6	24.4	23.9	22.0	21.2	13.4	8.0

**ANOVA**																							
CO_2_		**	ns			**			**			+			**			*			**		
P		−	**			**			**			**			**			**			**		
LC3		ns	ns			ns			**			**			**			**			**		
CO_2_ × P		−	ns			ns			ns			+			ns			ns			ns		
CO_2_ × LC3		ns	ns			ns			ns			*			**			ns			*		
P × LC3		−	ns			ns			**			**			**			**			**		
CO_2_ × LC3 × P		−	ns			ns			ns			ns			*			ns			ns		

AC, ambient CO_2_; EC, elevated CO_2_ (AC + 200 ppm). LC, leaf-cutting treatment; CK, control, no leaf-cutting; LC3, cutting off top three leaves. P, position; SS, superior spikelet; MS, medium spikelet; IS, inferior spikelet. −, data not available. **P < 0.01, *P < 0.05, ^+^0.05 < *P* < 0.1. *n* = 3.

Similar to EXP1 results, eCO_2_ and LC3 had substantial effects on FGP and EGP but little effects on IGP (Table 4). Compared with ambient CO_2_, eCO_2_ significantly increased FGP by 3.4% on average; among the different SSTs, CK and LC3 crops were significantly increased by 1.7 and 6.5%, respectively. Among the different grain positions, the responses of FGP of the middle and lower grain were slightly greater than those of the upper grain. Compared with CK, LC3 significantly decreased the FGP by 43.1% on average, and the FGP values of SS, MS, and IS were significantly decreased by 19.8, 51.4, and 67.2%, respectively. The responses of FGP were mainly related to EGP: compared with ambient CO_2_, eCO_2_ significantly reduced the EGP by about 13.2%, and the LC3 crop was reduced more significantly than CK, as indicated by the significant CO_2_ × LC3 interaction. Compared with CK, LC3 significantly increased the EGP, and the increase of MS and IS was greater than that of SS, which led to a significant LC3 × position interaction.

The response of FGW to eCO_2_ and LC3 was lower than that of AGW (Table 4). Compared with ambient CO_2_, eCO_2_ significantly increased FGW and AGW by 1.9 and 4.8%, respectively, and the response of LC3 crops was more pronounced than that of CK, whereas that of IS was slightly higher than that of SS or MS. For instance, eCO_2_ increased AGW of CK and LC3 crops by 1.9 and 9.7%^∗∗^ and significantly increased that of SS, MS, and IS by 4.4, 5.0, and 5.5%, respectively. Compared with CK, LC3 significantly decreased FGW and AGW by about 5.7 and 37.3%, respectively, and the response trend of AGW followed the order IS (53.9%^∗∗^) > MS (43.0%^∗∗^) > SS (21.9%^∗∗^); for FGW, the opposite trend was observed. There was no interaction effect between CO_2_ and LC3 on AGW, whereas position × LC3 had a significant impact on both parameters.

### Biomass Accumulation and Distribution at Maturity

**FIGURE 4:**
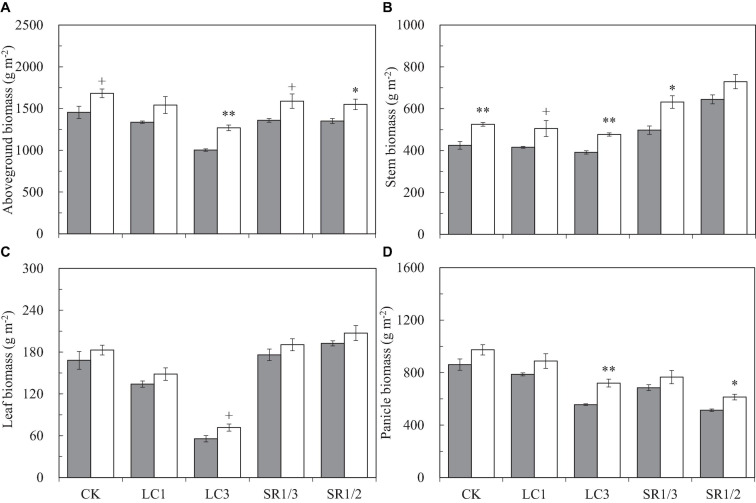
Aboveground biomass **(A)**, stem biomass **(B)**, leaf biomass **(C)**, and panicle biomass **(D)** of WYJ27 per unit area at maturity affected by elevated CO_2_ and different source-sink treatments in 2017. Each bar in the figure represents the mean values across three plots for ambient CO_2_ (aCO_2_, filled square) or elevated CO_2_ (eCO_2_, aCO_2_ + 200 ppm, unfilled square); vertical bars represent standard error (*n* = 3). CK, no leaf cutting or spikelet removing; LC1, cutting off the flag leaf; LC3, cutting off top three leaves; SR1/3, removing one branch in every three branches of a panicle; SR1/2, removing one branch in every two branches of a panicle. **P < 0.01, *P < 0.05, ^+^*P* < 0.1.

Compared with ambient CO_2_, eCO_2_ significantly increased the aboveground biomass (AGB) by 17.3%, and the response of LC3 crops (27%) was almost twice that of SR1/2 crops (15%) (Figure 4). Compared with CK, LC and SR significantly decreased AGB; among the different SSTs, LC3 crops had the largest decrease (28%), and the decreases caused by other SSTs were similar (about 7%). Compared with ambient CO_2_, eCO_2_ significantly increased the dry weights of stem, leaf, and panicle by 20.8, 10.3, and 16.4%, respectively. The increase in stem weight was similar under different SSTs; however, the response of leaf and panicle to eCO_2_ under LC3 was significantly higher than that under other SSTs. For example, eCO_2_ increased the leaf weight of CK, LC1, LC3, SR1/3, and SR1/2 crops by 8.8, 10.8, 28.9^+^, 8.2, and 7.7%, respectively. Compared with CK, cutting leaves reduced the dry weight of each organ uniformly, but the effect of spikelet removal was different in various organs; for instance, SR1/3 and SR1/2 significantly increased stem weight by 18.8 and 44.5% and leaf weight by 4.4 and 13.8%^∗^, but significantly reduced panicle weight by 20.9 and 38.6%, respectively. There was no interaction between CO_2_ and SSTs on AGB or its components.

Compared with ambient CO_2_, eCO_2_ significantly increased the dry weight ratio of the stem (RS) by about 2.6% and decreased the dry weight ratio of the leaf (RL) by 5.1% (Supplementary Figure 4); it had no significant effect on the dry weight ratio of the panicle (RP). Among the crops under different SSTs, eCO_2_ significantly increased the RS of CK, LC1, and SR1/3 crops by 6.9, 5.2, and 8.8%, respectively, and slightly decreased the RS of LC3 and SR1/2 crops. No significant influence of eCO_2_ was observed on RL or RP of crops under different SSTs (Supplementary Figures 4B,C). Compared with CK, spikelet removal significantly increased RL, especially for RS (26.3^∗∗^ and 56.6%^∗∗^ by SR1/3 and SR1/2, respectively), but decreased RP (15.8^∗∗^ and 33.7%^∗∗^ by SR1/3 and SR1/2, respectively). However, leaf cutting decreased RL but increased RS, resulting in a negligible effect on the RP (Supplementary Figure 4C). Analysis of variance showed that CO_2_ × LC3 and CO_2_ × SR1/2 had significant impacts on RS and RP (Table 1).

### NSC Content of the Stem at Different Periods After Heading

**FIGURE 5:**
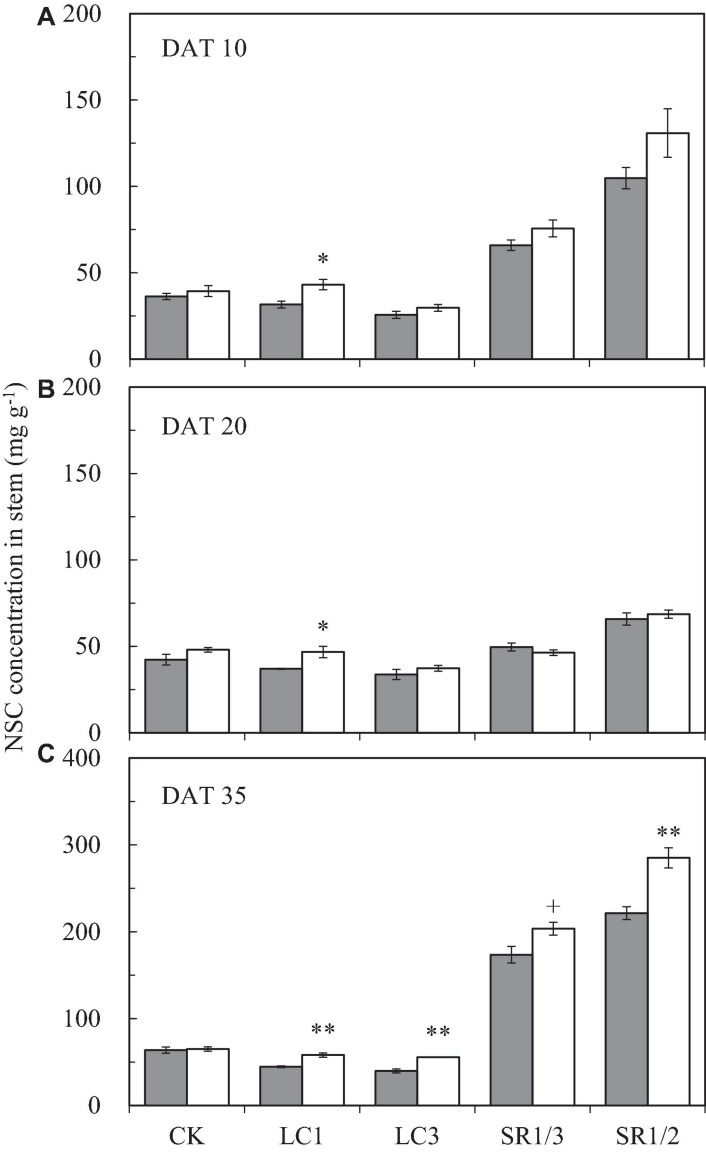
The NSC concentration in stem of WYJ27 at DAT10 **(A)**, DAT20 **(B)**, and DAT35 **(C)** affected by elevated CO_2_ and different source-sink treatments in 2017. Each bar in the figure represents the mean values across three plots for ambient CO_2_ (aCO_2_, filled square) or elevated CO_2_ (eCO_2_, aCO_2_ + 200 ppm, unfilled square); vertical bars represent standard error (*n* = 3). CK, no leaf cutting or spikelet removing; LC1, cutting off the flag leaf; LC3, cutting off top three leaves; SR1/3, removing one branch in every three branches of a panicle; SR1/2, removing one branch in every two branches of a panicle. DAT, day after source-sink treatment. **P < 0.01, *P < 0.05, ^+^*P* < 0.1.

The NSC content of the stem at different stages followed the order DAT35 > DAT10 > DAT20 (DAT, day after SST) (Figure 5). Compared with ambient CO_2_, eCO_2_ significantly increased the NSC content by 19.1% on average during the grain filling stage. Among the different SSTs, CK, LC1, LC3, SR1/3, and SR1/2 crops were increased by 7.1, 30.7^∗∗^, 23.7^∗∗^, 12.6^∗∗^, and 23.6%^∗∗^, respectively; among the different stages, eCO_2_ significantly increased the NSC content by 20.6, 8.2, and 22.9% at DAT10, DAT20, and DAT35, respectively. Compared with CK, LC1 and LC3 significantly reduced the NSC content by 11.4 and 24.8%, respectively. On the contrary, removing spikelets significantly increased the NSC content of the stem, especially at DAT35. Different degrees of the effects of interaction between CO_2_ and SSTs on the NSC content of the stem at DAT35 were observed (Table 5).

**TABLE 5:** Significance test for the NSC concentration in stem of WYJ27at DAT10, DAT20, and DAT35 affected by elevated CO_2_ and different source-sink treatments in 2017.

Stage	CO_2_	LC1	LC3	SR1/3	SR1/2	CO_2_ × LC1	CO_2_ × LC3	CO_2_ × SR1/3	CO_2_ × SR1/2
DAT 10	**↑	ns	**↓	**↑	**↑	ns	ns	ns	ns
DAT 20	*↑	ns	**↓	ns	ns	ns	ns	+	ns
DAT 35	**↑	**↓	**↓	**↑	**↑	*	*	+	**

LC1, cutting off the flag leaf; LC3, cutting off top three leaves; SR1/3, removing one branch in every three branches of a panicle; SR1/2, removing one branch in every two branches of a panicle. DAT, day after source-sink treatment. **P < 0.01, *P < 0.05, ^+^*P* < 0.1; ns, not significant; *n* = 3. Arrows in the treatment column indicate treatment increased (↑) or decreased (↓) the values.

### Leaf *Pn* at Different Periods After Heading

**FIGURE 6:**
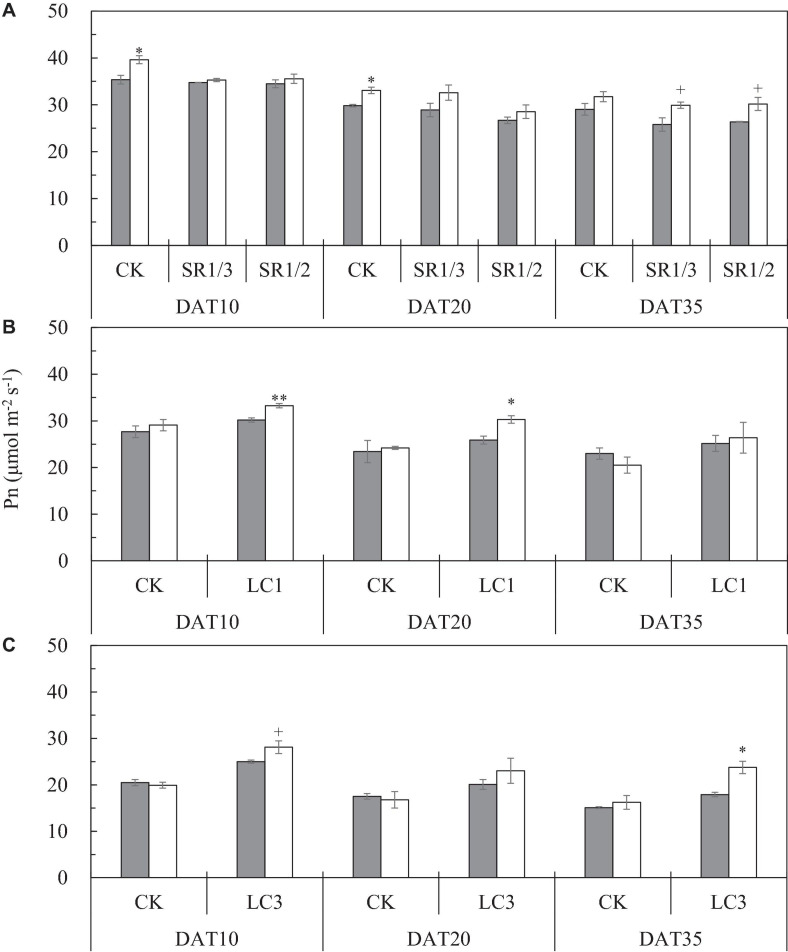
The Pn of flag leaf [**(A)**; CK, SR1/3, and SR1/2], second leaf [**(B)**; CK and LC1], and fourth leaf [**(C)**; CK and LC3] of WYJ27 at different stages affected by elevated CO_2_ and different source-sink treatments in 2017. Each bar in the figure represents the mean values across three plots for ambient CO_2_ (aCO_2_, filled square) or elevated CO_2_ (eCO_2_, aCO_2_ + 200 ppm, unfilled square); vertical bars represent standard error (*n* = 3). SR, spikelet removing treatments; CK, no leaf cutting or spikelet removal; LC1, cutting off the flag leaf; LC3, cutting off top three leaves; SR1/3, removing one branch in every three branches of a panicle; SR1/2, removing one branch in every two branches of a panicle. DAT, day after source-sink treatment. **P < 0.01, *P < 0.05, ^+^*P* < 0.1.

We observed that the *Pn* of the flag leaf gradually decreased with the growth stage. Compared with ambient CO_2_, eCO_2_ significantly increased the *Pn* of the flag leaf by 9.3% on average during the grain filling stage (Figure 6A). The response of SR crops to eCO_2_ at DAT10 and DAT20 was less pronounced than that of CK. Compared with CK, SR1/3 and SR1/2 significantly reduced the *Pn* of the flag leaf by 5.7 and 8.5% on average over the three stages, respectively. However, no interaction was observed among CO_2_, SR, and stage (Table 6).

**TABLE 6:** Significance test for the *Pn* of flag leaf (CK, SR1/3, and SR1/2 crops), second leaf (CK and LC1 crops) and fourth leaf (CK and LC3 crops) of WYJ27 at different stages as affected by elevated CO_2_ and different source-sink treatments in 2017.

Leaf position	CO_2_	SST	Stage	CO_2_ × SST	CO_2_ × Stage	Stage × SST	CO_2_ × Stage × SST
The flag leaf	**↑	**↓	**	ns	ns	ns	ns
The second leaf	ns	**↑	**	ns	ns	ns	ns
The fourth leaf	*↑	**↑	**	*	ns	ns	ns

CK, no leaf cutting or spikelet removing; LC1, cutting off the flag leaf; LC3, cutting off top three leaves; SR1/3, removing one branch every three branches of a panicle; SR1/2, removing one branch every two branches of a panicle. SST, source-sink treatment. DAT, day after source-sink treatment. **P < 0.01, *P < 0.05; ns, not significant; *n* = 3. Arrows in the treatment column indicate treatment increased (↑) or decreased (↓) the values.

To compare the *Pn* values of the same leaf of CK and LC1 crops, we measured *Pn* of the second leaf, which also gradually decreased during the grain filling stage (Figure 6B). Averaged over two SSTs (CK and LC1) and three determination stages, eCO_2_ increased the *Pn* of the second leaf by 5.4%, and this was mainly related to the significant increase of LC1 crops (10.7%), whereas CK crops showed no significant response. Compared with CK, the *Pn* of LC1 crops showed an advantageous response to eCO_2_, especially at the early and middle stages of grain filling. Compared with CK, LC1 significantly increased the *Pn* of the second leaf during the grain filling stage by 15.7%. There was no interaction effect among CO_2_, LC1, and stage on the *Pn* of the second leaf.

We measured the *Pn* of the fourth leaf of CK and LC3 crops to compare the *Pn* values of the same leaf position. The *Pn* of the fourth leaf gradually decreased during the grain filling stage (Figure 6C). Compared with ambient CO_2_, eCO_2_ significantly increased the *Pn* of the fourth leaf by 10.1% on average, and that of LC3 crops was significantly increased by 18.9%; in contrast, CK was not affected. The response of LC3 crops in the FACE plots was more apparent at DAT35, indicating that LC3 rice could weaken or prevent photosynthetic adaptation at the later stage of grain filling. Compared with CK, LC3 significantly increased the *Pn* of the fourth leaf by about 30% on average. A significant interaction between CO_2_ and LC3 was observed on the *Pn* of the fourth leaf.

## Discussion

### Impact of Source-Sink Relationships on Grain Yield and Its Components

We showed that a CO_2_ concentration elevated by 200 ppm in a FACE system resulted in similar yield increases for naturally grown (CK) rice in both seasons (14% in 2017 and 16% in 2018). This was comparable to the yield increase for *japonica* rice in a recent integrated analysis of FACE studies (12.7%) (Hu et al., 2021). However, little is known about the alterations in source-sink relations and their effects on rice yield responses to eCO_2_. Therefore, different leaf-cutting or spikelet removal treatments were used at the heading stage to change the original source-sink relationship of rice. The EXP1 showed that yield increases of LC1 (18%) and LC3 (25%) crops were higher than that of CK when the CO_2_ level was increased to 580 ppm. The more leaves were cut, the more significantly increased the yield response; however, this trend was slightly decreased under SR1/3 and SR1/2. Only the top three leaves were cut off in 2018 (EXP2), and the yield increase of LC3 crops by eCO_2_ was about 10% higher than that of CK (Figure 3). These results indicate that changing the source-sink relationship artificially had a relatively significant regulatory effect on the response of yield to eCO_2_. Reviews (Wang et al., 2015; Hu et al., 2021) have shown that the growth and yield responses of *hybrid* and *indica* rice under eCO_2_ are significantly higher than those of *japonica* rice. Therefore, whether human-induced source-sink regulation affects the yield response of *indica* or *hybrid* rice to eCO_2_ needs to be investigated in further studies.

A meta-analysis of FACE studies (Hu et al., 2021) has shown that the increase of rice yield under eCO_2_ is mainly related to the increase of PNA and FGP, whereas SNP or FGW do not change significantly. We verified these results for two seasons. The changes in IGP and EGP suggest that the response of FGP to eCO_2_ was mainly caused by the change of EGP. Except for removing spikelets, which significantly reduced SNP, all main factors (CO_2_ and SSTs) and their interactions had no significant impacts on PNA or SNP. Most likely, this was because the process of rice tillering and spikelet formation had finished before SST, indicating that the samples selected for the SST were adequately represented in this study.

The main purpose of artificial SST at the heading stage was to observe any changes in grain filling capacity, which was generally expressed by FGP and FGW. The responses of FGP of LC1 and LC3 crops in the FACE plots were more than two and three times the CK, which were comparable to the yield, whereas SR1/3 and SR1/2 crops showed only slight changes. The AGW also reflected fertilization and grain filling, the responses of which to eCO_2_ and SSTs were similar to that of FGP. Correlation analysis also showed that the yield response to eCO_2_ was significantly positively correlated with FGP and AGW, indicating that adjustment by SST to the final yield response is mainly related to the response of AGW, especially FGP. In the present study, the starting time of treatment of FACE was after seedling transplanting, while the specific effect of CO_2_ treatment time on the results is worthy of further study.

By separating grains into SS, MS, and IS, corresponding to spikelets located at the upper, middle, and lower parts of a panicle, respectively, we analyzed the effects of eCO_2_ and high-intensity leaf cutting (LC3) on the yield components in EXP2. Generally, SS responded only slightly to environmental changes, whereas MS and IS were more sensitive. This has been reported in previous studies about rice under high-temperature stress (Mohammed and Tarpley, 2010), CO_2_-enrichment (Hu et al., 2019), and ozone-inducement (Shao et al., 2020). Our results also indicate that eCO_2_ increased the yield of grains located at different positions in a panicle, following the order IS > MS > SS; this was more obvious for LC3 crops. Similarly, this order was also observed for the yield reduction by LC3 compared with CK (Figure 3). As mentioned above, different responses of yield to different conditions are mainly related to FGP and AGW. The effects of eCO_2_ or LC3 on these two parameters are shown as an SS less than MS or IS. Therefore, the grain-filling capacity of the middle and lower panicles has a greater plasticity. Greater plasticity in response to a changing climate, especially in resource-rich (increasing CO_2_) environments, can provide advantages over plants with low plasticity (Kikuchi et al., 2017). Exploiting the filling capacity of these grains under eCO_2_ is essential to maximize eCO_2_ use.

Our results suggest that eCO_2_ significantly increased the NSC content of the stem, which was comparable to the results of a previous FACE study by Zhu et al. (2016), indicating that the increase in atmospheric CO_2_ concentration significantly promotes rice growth, leading to an AGB increase (Wang et al., 2020). From the point of view of biomass accumulation and distribution, eCO_2_ had no significant influence on the dry weight ratio of the panicle for crops under different SSTs; therefore, the yield increase under eCO_2_ is mainly related to the increase of photosynthetic production capacity, whilst the distribution of photosynthetic products to panicles does not change.

### Source-Sink Relationships Affect Sugar Accumulation and Photosynthesis Under eCO_2_

The positive response of leaf net photosynthesis to eCO_2_ is essential for rice growth and yield increase (Cai et al., 2020). Previous studies have shown that eCO_2_ can increase the net photosynthesis in the short term (Drake et al., 1997). However, crops growing under eCO_2_ over a long period can achieve photosynthetic adaptation or down-regulation (Zhu et al., 2014). In the present study, eCO_2_ significantly increased flag leaf *Pn* of naturally growing (CK) crops at the early stage of grain filling. However, this gradually decreased over time. Obviously, the photosynthetic adaptation is not conducive to increase the rice yield potential under eCO_2_. There is no consensus on why this happens and how to reduce or avoid it. In our study, the *Pn* response of flag leaf to eCO_2_ of the spikelet removal crops was weakened, especially at DAT10 and DAT20, compared with that of CK. Because photosynthesis measurements were not made at the same time during the day, this may have affected the comparison of *Pn* between treatments. However, this effect may be small; for example, in some previous studies (Shao et al., 2014; Yuan et al., 2018), the *Pn* values in the morning were similar to those in the afternoon over the measuring period we selected. The mitigation of the photosynthesis level for the SR treatment under eCO_2_ was due to the increase in NSC in the leaves (Supplementary Figure 5 and Supplementary Table 1). Fabre et al. (2020) also pointed out that low source-sink ratio cultivars had greater gains in photosynthesis because they accumulated less NSCs in the flag leaf than high source-sink ratio cultivars. Conversely, leaf-cutting treatments (LCs) enhanced the *Pn* response of the remaining leaves to eCO_2_ (i.e., LC1 enhanced *Pn* of the second leaf and LC3 enhanced *Pn* of the fourth leaf). This phenomenon was observed throughout the whole grain-filling period. Spikelet removal resulted in a significant decrease in dry weight ratio of the panicle, which is consistent with Shimono et al. (2010). The proportion of the ‘sink’ is significantly smaller than that of the ‘source,’ increasing the ratio of source to sink. Crops with a high source-sink ratio waste the ‘source’ supply under eCO_2_, since part of the photosynthate cannot be transferred to the grain. Stems are sites of temporary carbon storage that can be remobilized to reproductive tissues, significantly contributing to grain filling in later developmental stages (Hirose et al., 2006). However, for crops with high SSTs (spikelet removal), the carbon may not be remobilized to compensate for a lack of photosynthesis during the grain filling phase under eCO_2_ (MacNeill et al., 2017; Fabre et al., 2020), potentially causing a feedback inhibition effect on the net photosynthetic rate of rice (Ding et al., 2005). Our findings suggest that the *Pn* of flag leaf was significantly lower than that of CK because of the removal of spikelets, especially at the beginning of the grain-filling period. Fabre et al. (2019) also indicate that the *Pn* of crops with pruning treatment (a total removal of the panicles) is lower than that of CK, and this phenomenon is more pronounced under eCO_2_. In our study, plants with low SSTs (leaf cutting) showed an increase in NSC reserves under eCO_2_, probably because these plants mobilized stem reserves less exhaustively under eCO_2_ for grain filling. Plants therefore do not rely on these reserves because of the greater level of photosynthesis. The LCs decreased the sources-sink ratio, and the grain was ‘hungry’ due to insufficient assimilate supply during grain filling. Plants will determine the priority of assimilate acquisition among various organs to achieve balanced growth and development (Lemoine et al., 2013). Therefore, assimilates may preferably be supplied to the grain, and part of the stem NSC is consumed to increase the grain supply at the same time. Crops with a low source-sink ratio can reduce the excessive accumulation of assimilation substances in the stem, which is more suitable for the continuous extraction of assimilates from ‘source’ organs to ‘sink’ organs, improving the response of *Pn* to eCO_2_. In addition, this also occurs at the later stage of grain filling, indicating that photosynthetic adaptation can be weakened or even eliminated. As we cannot really decide on the role of sugars in the present study, more research is needed on the regulation of carbohydrate accumulation and remobilization in the context of source-sink relationships.

Fabre et al. (2020), using five naturally contrasting cultivars in terms of their source-sink ratio to investigate the response of photosynthetic capacity to eCO_2_, found a negative correlation between source-sink ratio and the response of *Pn*. Therefore, compared with a high source-sink ratio, a low source-sink ratio may be more conducive to improve photosynthetic capacity and maximize the ‘fertilizer effect’ of eCO_2_. Similar phenomena have been observed for other C_3_ plants. For example, high sink-strength varieties of tobacco (Ruiz-Vera et al., 2017) and cassava (Ruiz-Vera et al., 2020) could prevent the photosynthetic down-regulation of leaves under FACE conditions. Nitrogen availability by the plant is an essential factor that can explain the variations of photosynthesis (Wang et al., 2015; Ruiz-Vera et al., 2017). It will be also important to consider this factor in future studies that we could not evaluate here. Our study not only serves as a reference for breeders (see also Dingkuhn et al., 2020), but also indicates that the source-sink ratio of rice can be appropriately adjusted by agronomic management practices against the background of increasing CO_2_ levels.

## Conclusion

Artificial reduction of the source-sink ratio (such as cutting off leaves) could enhance the ‘fertilizer effect’ of CO_2_, whereas increasing the source-sink ratio (such as removing spikelets) had a tendency to weaken the ‘fertilizer effect.’ The former was mainly related to the increase of the responses of FGP and AGW to eCO_2_, and the latter decreased those responses. Removing spikelets increased the source-sink ratio and significantly increased the NSC content of the stem compared with CK, which resulted in a feedback inhibition to enhance the photosynthetic adaptation and reduce the final yield increase under eCO_2_. Conversely, cutting off leaves, which reduced the source-sink ratio, could match the sink strength by increasing the net photosynthesis of the remaining leaf to enhance the yield increase caused by eCO_2_. Our findings suggest that the source-sink ratio of a field population can be reduced by appropriate agronomic management (such as using flower fertilizer to promote spikelet differentiation to enlarge the sink capacity, the use of breeding cultivars with a lower ratio of source to sink, controlling ineffective tillers, and reducing ineffective and inefficient leaf area by water management) to achieve a higher level of source-sink balance in a CO_2_-rich environment, which will be more conducive to maximizing the ‘fertilizer effect’ of CO_2_. Since only one *japonica* rice variety was used in this study, further studies are needed to determine whether these findings can be extrapolated to other varieties, such as *indica* or *hybrid* rice.

## Data Availability Statement

The original contributions generated for this study are included in the article/Supplementary Material, further inquiries can be directed to the corresponding author.

## Author Contributions

BG: conceptualization, methodology, data curation, investigation, and writing – original draft. SH, LJ, and XN: data curation. YXW: supervision, validation, and funding acquisition. JZ: supervision and validation. YLW: supervision and writing – review and editing. LY: conceptualization, methodology, writing – review and editing, supervision, and funding acquisition. All authors contributed to the article and approved the submitted version.

## Conflict of Interest

The authors declare that the research was conducted in the absence of any commercial or financial relationships that could be construed as a potential conflict of interest.
