# Supplementary material for: Alterations in Source-Sink Relations Affect Rice Yield Response to Elevated CO2: A Free-Air CO2 Enrichment Study
Source: Front Plant Sci. 2021 Jul 2;12:700159. doi: 10.3389/fpls.2021.700159 (PMC8283783; doi:10.3389/fpls.2021.700159)
Supplement: Supplementary file 1 [file Data_Sheet_1.docx]

**Supplementary materials:**

**Fig. S1.** Maximum (red line), minimum (blue line), and mean (black line) daily temperature during 2017 (a) and 2018 (b) growing seasons.

**Fig. S2.** Spikelet number per panicle (a), total spikelet number per area (b), fully-filled grain weight (c) and average grain weight of all seeds (d) of WYJ27 affected by elevated CO_2_ and different source-sink treatments in 2017. Each bar in the ﬁgure represents the mean values across three plots for ambient CO_2_ (aCO_2_, filled square) or elevated CO_2_ (eCO_2_, aCO_2_ + 200 ppm, unfilled square); vertical bars represent standard error (n = 3). CK, no leaf cutting or spikelet removing; LC1, cutting off the flag leaf; LC3, cutting off top three leaves; SR1/3, removing one branch in every three branches of a panicle; SR1/2, removing one branch in every two branches of a panicle. ^*^ *P* < 0.05; ^+^ *P* < 0.1.

**Fig. S3.** Relationships between CO_2_-induced changes in grain yield and filled-grain rate (a, c) and average grain weight of all seeds (b, d) of WYJ27. CK, no leaf cutting or spikelet removing; LC1, cutting off the flag leaf; LC3, cutting off top three leaves; SR1/3, removing one branch in every three branches of a panicle; SR1/2, removing one branch in every two branches of a panicle. Each point is an average per source-sink treatment ± standard error (n = 3).

**Fig. S4.** The ratio of stem (a), leaf (b), and panicle (c) dry weight of WYJ27 at maturity affected by elevated CO_2_ and different source-sink treatments in 2017. Each bar in the ﬁgure represents the mean values across three plots for ambient CO_2_ (aCO_2_, filled square) or elevated CO_2_ (eCO_2_, aCO_2_ + 200 ppm, unfilled square); vertical bars represent standard error (n = 3). CK, no leaf cutting or spikelet removing; LC1, cutting off the flag leaf; LC3, cutting off top three leaves; SR1/3, removing one branch in every three branches of a panicle; SR1/2, removing one branch in every two branches of a panicle. ^*^ *P* < 0.05; ^+^ *P* < 0.1.

**Fig. S5.** The NSC concentration in leaf of WYJ27 at DAT10 (a), DAT20 (b), and DAT35 (c) affected by elevated CO_2_ and different source-sink treatments in 2017. Each bar in the ﬁgure represents the mean values across three plots for ambient CO_2_ (aCO_2_, filled square) or elevated CO_2_ (eCO_2_, aCO_2_ + 200 ppm, unfilled square); vertical bars represent standard error (n = 3). CK, no leaf cutting or spikelet removing; LC1, cutting off the flag leaf; LC3, cutting off top three leaves; SR1/3, removing one branch in every three branches of a panicle; SR1/2, removing one branch in every two branches of a panicle. DAT, day after source-sink treatment. ^*^ *P* < 0.05; ^+^ *P* < 0.1.

**Table S1**

Significance test for the NSC concentration in leaf of WYJ27 at DAT10, DAT20, and DAT35 under elevated CO_2_ and different source-sink treatments in 2017.

| Stage | CO_2_ | LC1 | LC3 | SR1/3 | SR1/2 | CO_2_×LC1 | CO_2_×LC3 | CO_2_×SR1/3 | CO_2_×SR1/2 |
| --- | --- | --- | --- | --- | --- | --- | --- | --- | --- |
| DAT 10 | **↑ | *↓ | *↓ | ns | ns | ns | * | ns | ns |
| DAT 20 | **↑ | *↓ | **↓ | ns | *↑ | ns | ns | ns | ns |
| DAT 35 | **↑ | **↓ | ns | +↑ | **↑ | ns | ns | ns | ns |

LC1, cutting off the flag leaf; LC3, cutting off top three leaves; SR1/3, removing one branch in every three branches of a panicle; SR1/2, removing one branch in every two branches of a panicle. ^**^ *P* < 0.01; ^*^ *P* < 0.05; ^+^ *P* < 0.1; ns, not significant. Arrows in the treatment column indicate treatment increased (↑) or decreased (↓) the values.
